# *Drosophila melanogaster* as a Model for Diabetes Type 2 Progression

**DOI:** 10.1155/2018/1417528

**Published:** 2018-04-24

**Authors:** Jéssica P. Álvarez-Rendón, Rocío Salceda, Juan R. Riesgo-Escovar

**Affiliations:** ^1^Instituto de Neurobiología, Universidad Nacional Autónoma de México, Campus UNAM Juriquilla, Boulevard Juriquilla No. 3001, 76226 Querétaro, QRO, Mexico; ^2^Instituto de Fisiología Celular, Universidad Nacional Autónoma de México, Avenida Universidad No. 3000, Colonia Universidad Nacional Autónoma de México, Delegación Coyoacán, 04510 Ciudad de México, Mexico

## Abstract

*Drosophila melanogaster* has been used as a very versatile and potent model in the past few years for studies in metabolism and metabolic disorders, including diabetes types 1 and 2.* Drosophila *insulin signaling, despite having seven insulin-like peptides with partially redundant functions, is very similar to the human insulin pathway and has served to study many different aspects of diabetes and the diabetic state. Yet, very few studies have addressed the chronic nature of diabetes, key for understanding the full-blown disease, which most studies normally explore. One of the advantages of having* Drosophila* mutant viable combinations at different levels of the insulin pathway, with significantly reduced insulin pathway signaling, is that the abnormal metabolic state can be studied from the onset of the life cycle and followed throughout. In this review, we look at the chronic nature of impaired insulin signaling. We also compare these results to the results gleaned from vertebrate model studies.

## 1. Introduction

Diabetes is a chronic metabolic malaise that affects and is forecast to affect many millions of people in the world [1]. It is a disease caused by insulin deficiency or loss of insulin action. In addition to genetic factors, certain lifestyles such as high dietary fat content and physical inactivity are risk factors for the development of diabetes [2]. It has outpaced many other diseases and is predicted to become one of the major health concerns in the future [3]. According to data cited by the World Health Organization, by 2014 incidence of diabetes had risen to 8.5% [3]. In Mexico, for example, 2017 figures show that over 15% of adults are diabetic, which is a very high incidence and concern [4]. As of now, diabetes is an incurable and incapacitating disease with a long and protracted progression. It is also a disease being diagnosed more often in younger patients [2].

In human diabetic patients where the condition has existed for some time, there are several comorbidities. It courses with macrovascular complications, leading to heart disease and stroke, and increased cardiovascular morbidity and mortality. In addition, microvascular complications lead to nephropathy, retinopathy, and neuropathy [1]. Little is known of the onset and early progression of the disease, except for familial cases, which are the minority, and the higher risk of diabetes type 2 for babies where mothers had hyperglycemia or diabetes [2, 5].

Diabetes mellitus is divided into basically two types: type 1 and type 2, a division that reflects the cause of the metabolic dysfunction. Diabetics type 1 have a reduction in insulin secretion, and as a consequence, blood glucose does not attain homeostatic levels after food ingestion and digestion. Physicians normally treat them by prescribing exogenous insulin injections on a regular basis. These diabetics represent around 10% of all diabetic patients, and in most cases, their condition is due to the death of pancreatic Langerhans islets ß-type cells, which normally secrete insulin to clear elevated glucose levels from the bloodstream, like after a meal [6]. It leads to elevated blood glucose levels, as expected, and to general body wasting.

Diabetes type 2 represents the majority of cases, ranging between 90 and 95% of all diabetic patients. It is characterized by a combination of insulin resistance and insulin secretion defects, resulting in relative insulin deficiency and hyperglycemia [6]. Diabetic type 2 patients normally represent patients that have had a long progression, initially suffering from metabolic syndrome, and/or being overweight, and/or being obese for several years. Environmental factors, like diet and level of physical exercise, also play an important role in the inception and progression of the disease, as noted above.

Finally, there is also a third type of diabetes: gestational diabetes. This form of diabetes occurs in pregnant women, leads to increased risk of diabetes for the offspring, and may lead to diabetes type 2 in the mothers after birth [2].

There are, in sum, many factors causing diabetes type 2, both genetic and environmental, and the composite picture is complex, as it may change depending on the actual combination present in populations and individual patients [2]. While all of the factors cited above are recognized contributing factors, it is not clear how they weigh in the initiation and early progression of the disease. Therefore, it is important to elucidate the precise molecular mechanisms underlying the development and progression of the disease.

**Figure 1:**
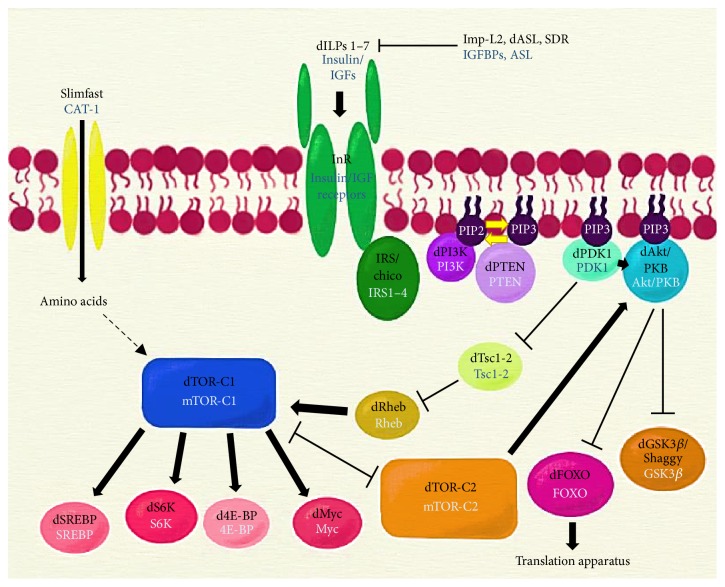
The insulin signaling pathway. The binding of insulin to its receptor initiates a phosphorylation cascade that results in the regulation of metabolism through several effectors. Names for the vertebrate counterparts of the pathway appear below their* Drosophila *names. CAT-1: cationic amino acid transporter-1; Imp-L2: ecdysone-inducible gene L2; IGFBPs: insulin-like growth factor binding proteins; ASL: acid-labile subunit; SDR: secreted decoy of InR; dILPs 1–7: insulin-like ligands 1–7; IGFs: insulin-like growth factors; InR: insulin receptor; IRS/chico: insulin receptor substrate; PI3K: phosphatidylinositol 3-kinase (two subunits: Pi3K92E is the catalytic subunit, and Pi3K21B is the regulatory subunit); PIP2: phosphatidylinositol 4,5-bisphosphate; PIP3: phosphatidylinositol 3,4,5-trisphosphate; PTEN: phosphatase and tensin homolog; dPDK1: 3-phosphoinositide dependent protein kinase-1; GSK3*β*: glycogen synthase kinase 3 beta; Tsc1-2: tuberous sclerosis proteins 1 and 2; Rheb: Ras homolog enriched in brain; TOR-C1: target of rapamycin complex 1 (the TOR-C1 complex consists primarily of TOR, regulatory associated protein of TOR (raptor), and lethal with Sec-13 protein 8 (LST8)); TOR-C2: target of rapamycin complex 2 (the TOR-C2 complex consists primarily of TOR, rapamycin-insensitive companion of TOR (Rictor), and stress-activated protein kinase-interacting protein 1 (Sin1)); Myc: Myc protein; SREBP: sterol regulatory element-binding protein; S6K: ribosomal protein S6 kinase beta-1; 4E-BP: eukaryotic translation initiation factor 4E-binding protein 1; FoxO: Forkhead box O transcription factor. Dashed lines indicate an indirect interaction; arrows and bar-headed lines indicate activation and inhibition, respectively.

In general, the diabetic state is multifactorial encompassing several origins and progressions. Studying its causes, effects, and consequences is paramount in the actual diabetes “epidemic,” but it is not easy or even possible to study many of these aspects using human patients as test subjects. Scientists have developed model systems where diabetes can be controlled to a higher extent, and in which experimental setups with a high degree of rigor and reproducibility can be used, with genetic uniformity, and highly controlled environments. Principles uncovered in these systems can then be applied in a more general fashion, as the insulin pathway and glucose control is a common, evolutionarily conserved mechanism in the animal kingdom (Figure 1).

### 1.1. The Insulin Pathway

Insulin is an anabolic hormone in glucose homeostasis in experimentally pancreatomized dogs [7] discovered by Banting and Best, who won the Nobel Prize for this discovery [8]. In general in vertebrates, insulin is secreted from pancreatic Langerhans islets ß-type cells in response to increased glucose levels. In some teleost fish, insulin is produced in Brockmann's bodies [9]. Secreted insulin in the bloodstream binds to membrane receptors, especially in muscle cells, and initiates a transduction cascade that leads to glucose internalization and an anabolic response. In invertebrates, the insulin molecule is slightly longer, has one more disulphide bridge, and is secreted from specialized neurons (insulin-producing cells, or IPC) and glia in the brain [10]. Recently, in a striking novel use, insulin-like peptides have been identified in the venom of certain* Conus* mollusks able to bind insulin receptor molecules and induce hypoglycemia in fish prey [11, 12].

Insulin is a small polypeptide constituted by two chains linked by disulphide bonds, synthesized from the same gene [13]. Whereas vertebrates have one insulin gene, the* Drosophila* genome codes for seven several insulin-like peptides, secreted from the insulin-producing cells (IPC) of the brain. A further eight* Drosophila *insulin-like peptide, DILP8, is really a relaxin homolog, binding to a different type of receptor, and controlling corporal symmetry [14–17].

The* Drosophila *insulin-like peptides (ILPs) also have nonredundant functions [18–20]. The ILP2 peptide has the highest homology to the vertebrate insulin gene and is synthesized together with ILP1, ILP3, and ILP5 in the IPCs of the brain, and their synthesis depends on ILP3. ILP3 expression also activates the insulin pathway in the fat body [21]. ILP4, ILP5, and ILP6 are expressed in the midgut, ILp7 is expressed in the ventral nerve chord, and ILp2 is also expressed in the salivary glands and imaginal discs [22]. The* Drosophila *IPCs are the equivalent of the mammalian Langerhans' islets ß pancreatic cells [23]. ILP6 is synthesized in the fat body and can partially substitute for ILP2 and ILP5. ILP2 loss-of-function mutations lead to an increase in lifespan, while loss in ILP6 causes reduced growth [23, 24].

An insulin monomer is around 50 amino acid residues in length, but dimers form in solution. Insulin is synthesized as a single polypeptide called preproinsulin, which is processed in the endoplasmic reticulum forming proinsulin, which then undergoes maturation through the action of peptidases releasing a fragment called the C-peptide and the A and B chains, linked by disulfide bonds. Mature insulin is exocytosed into the circulation by glucose stimulation and binds to plasma membrane receptors with tyrosine kinase activity.

Insulin is a potent anabolic hormone in vertebrates [25]. It also exerts a variety of actions in flies including effects on glucose, lipid, and protein metabolism. It directly promotes growth and proliferation in tissues, rather than differentiation [26, 27]. In vertebrates, insulin stimulates glucose uptake in skeletal muscle and fat, promotes glycogen synthesis in skeletal muscle, suppresses hepatic glucose production, and inhibits lipolysis in adipocytes [28]. Although vertebrate skeletal muscle, liver, and adipose tissue are considered the main target tissues of insulin action, there is evidence that insulin has important physiological functions in other tissues such as the brain, pancreas, heart, and endothelial cells [29, 30]. Pretty much the same is true for invertebrates in equivalent tissues, where insulin action has been shown to impinge on the physiology of many tissues, including the brain [31]. In vertebrates, there are insulin-growth factor binding proteins (IGFBPs) that conform to an evolutionarily conserved superfamily, regulating insulin-growth factor function; in* Drosophila* the homolog is* ecdysone-inducible gene 2 (Imp-L2)* [32].

In vertebrates, it is thought that insulin-like growth factor binding proteins, IGFBPs, and a third protein, ALS (acid-labile subunit), form ternary complexes with IGFs to regulate IGF function, separating insulin functions from IGFs functions [33]. In flies, ILPs have both vertebrate insulin and IGF functions. The* Drosophila *genome codes for a putative IGFBP-acid-labile subunit (IGFBP-ALS) homolog,* convoluted*, that has been shown to bind in vitro by ectopic expression to ILPs and Imp-L2 forming a ternary complex [34]. However, mutations (even null mutations) in* convoluted* have mutant phenotypes that differ from insulin pathway mutants.* Convoluted *mutants are larval lethal and affect tracheal morphogenesis and motor axon guidance. In addition,* convoluted* has a higher homology to extracellular matrix proteins like Chaoptin than to vertebrate ALS [35, 36]. Taken together, all of these facts cast doubt on whether a fly ALS homolog actually exists. It seems reasonable to postulate that since ILPs are both insulin and IGFs in flies, no separation in complexes is necessary.

Insulin or insulin-like peptides bind to the insulin receptor (IR), a heterotetrameric protein that consists of two extracellular *α*-subunits and two transmembrane *β*-subunits connected by disulfide bridges [37–40]. Insulin binding oligomerizes the receptors, allowing for cross-phosphorylation of the receptor molecules in tyrosine (tyr) residues in the IR domain of the intracellular part of the *β*-subunit. Despite some differences, vertebrate and invertebrate insulin receptors are equivalent [41], as chimeric fruit fly-vertebrate insulin receptors have been shown to be activated with a similar mechanism as vertebrate insulin receptors in mammalian cells [42]. In flies, there is also a secreted decoy of the insulin receptor,* secreted decoy of InR (Sdr)*, that binds some dILPs in circulation in the hemolymph, necessary for the negative regulation of Dilp action [43].

Phosphorylation in InR tyr residues in the intracellular part of the *β*-subunit, and the carboxy-terminal extension in the fruit fly insulin receptor [44], leads to the generation of protein binding sites. This leads to the subsequent recruitment, binding, and tyr phosphorylation of members of the insulin receptor substrate (IRS) family proteins [37]. In* Drosophila*, besides the carboxy-terminus extension of the insulin receptor, the IRS homologs* chico* [45] and* Lnk* [46] act as IRS type molecules. Whereas* chico *is the sole IRS homolog in flies [45],* Lnk* is the fly homolog of vertebrate SH2B adaptor proteins [47]. Lnk acts as an adaptor molecule that favors Chico and InR membrane localization [46].

The phosphorylated tyrosine residues in both the activated receptors and the IRS proteins create further binding sites for other molecules, like the catalytic subunit of phosphatidylinositol 3′ kinase (Pi3K92E), which via the regulatory subunit, Pi3K21B, can now be brought in proximity to its substrate, phosphatidylinositol (4,5) bisphosphate [48].

The phosphorylated residues of vertebrate IRS1 (or Chico, in the fruit fly) mediate an association with the SH2 domains of the p85 regulatory subunit of phosphatidylinositol 3-kinase (Pi3K) (Pi3k21B in flies [49]) leading to activation of the p110 catalytic subunit, which then catalyzes the formation of phosphatidylinositol (3,4, 5) trisphosphate (PI 3, 4, 5-P3) from phosphatidylinositol (4,5) bisphosphate (PI 4, 5-P2) in the inner leaflet of the plasma membrane [50]. This then creates binding sites for proteins with pleckstrin homology domains (PH) [51], like the phosphoinositide dependent kinase (PDK1) [52] and protein kinase B (PKB, also known as Akt) [53]. Both proteins bind, via their PH domains, the phosphatidylinositol trisphosphate generated in the inner membrane leaflet of the plasma membrane via action of Pi3K92E [54, 55]. PDK1, a serine-threonine kinase, then phosphorylates and activates Akt [56, 57].

The phosphorylating activity of Pi3K92E is counteracted by PTEN (phosphatase and tensin homolog deleted in chromosome ten), a lipid and protein phosphatase and tumor suppressor gene in vertebrates and flies [58, 59]. The lipid phosphatase activity, of phosphatidylinositol (3,4, 5) trisphosphate to phosphatidylinositol (4,5) bisphosphate is thought to be the main catalytic activity. It is deregulated in many tumor types in humans and in neurodegenerative diseases, like Parkinson's disease [60]. In* Drosophila*, another negative regulator of Pi3K92E is Susi, binding to the p60 regulatory subunit of PI3K, the 60 Kd molecular weight subunit [61, 62].

Akt/PKB is considered a critical node in insulin signaling. Akt/PKB acts by phosphorylating many different proteins [40]. In so doing, Akt/PKB activates different outcomes: (1) Glut 4-mediated glucose transport in vertebrates, by activating the protein Akt substrate of 160 kDa (AS160), (2) glycogen synthesis through inhibition of glycogen synthase kinase 3ß (GSKß), and hence, favoring glycogen synthase (GS) activity, (3) protein synthesis through the mammalian target of rapamycin (TOR) pathway, (4) inhibition of the Forkhead transcription factor FoxO, a major positive catabolic regulator, and (5) others targets, such as the SIK2 (salt-inducible kinase 2) [22, 63–66]. In* Drosophila,* Melted interacts with both FoxO and the TOR kinase via the tuberous sclerosis complex 2 protein (TSC2) and acts as a bridge within the insulin pathway regulating the activities of these two proteins [67].

Besides the direct effect on glucose metabolism, GSK3*β* also regulates cellular metabolism through the inhibitory regulation of transcription factors that globally control specific metabolic programs, and many of them are also regulated by TOR complexes: cell survival or proliferation (including c-Myc), the sterol regulatory element-binding proteins (SREBP1c), hypoxia-inducible factor 1-alpha (HIF1a), and the nuclear factor- (erythroid-derived 2-) like 2 (Nrf2) [68]. Thus, Akt signaling can stabilize these proteins by inhibiting GSK3*β* and by indirectly activating TOR-C1 [27]. There is evidence that the insulin pathway control of Myc is evolutionarily conserved in* Drosophila*. In biochemical experiments in tissue culture cells and in ectopic expression studies, the* Drosophila* insulin pathway, via inhibition of* shaggy, *the* Drosophila *homolog of GSK3*β*, regulated* Drosophila *Myc protein stability*. Drosophila myc *is coded by the gene* diminutive *[69, 70].

Perhaps the best-documented cases of downstream components activated by Akt/PKB are the target of rapamycin (TOR) kinase and the FoxO transcription factor. The ser/thr kinase TOR interacts with different proteins to form the complexes TOR-C1 and TOR-C2 [62, 71]. This kinase positively regulates cell growth, proliferation, motility, and survival. TOR-C1 appears to play a role in acute feedback inhibition of Akt, negatively regulating insulin action. Activation of TOR is not direct from Akt/PKB: in the* Drosophila* ovaries, Akt/PKB represses the proline-rich Akt substrate 40 kDa (PRAS40). There is also a PRAS40 homolog in vertebrates [72]. In the fly ovaries, PRAS40 represses TOR, decoupling reproduction from growth in the cells of this organ [73]. In other tissues, Akt/PKB phosphorylates and might repress TSC1 (in flies) and TSC2, which are normally thought to repress the GTP-binding protein and GTPase Rheb that activates TOR-C1, yet it is unclear whether indeed this is the case [74]. TOR-C1 activation leads to longer S1 phase in cells [75, 76].

TOR is another central component downstream of insulin signaling. The TOR kinase in the TOR-C1 complex phosphorylates and regulates several proteins. TOR kinase in the TOR-C1 complex phosphorylates (1) S6 kinase, to promote translation (S6 is a component of the ribosomes) [77], (2) the translation regulatory factor 4E-BP, which also promotes protein synthesis [78], (3) the transcription factor Myc [69, 79, 80], (4) SREBP [81], and (5) autophagy proteins (phosphorylation of these autophagy proteins represses them) [82]. TOR also regulates endocytosis to promote growth and repress catabolism [83]. Besides regulation by the insulin pathway, TOR-C1 is also regulated via a nutrient sensing signaling pathway, specifically via the activity of the amino acid transporter* Slimfast* [84].* dS6K* promotes ILP2 expression in the IPCs [21]. ILP secretion by IPCs is controlled by nutritional status, and this nutritional status is conveyed to IPCs by fat body cells, which secrete the Unpaired2 cytokine in fed conditions, which regulates GABAergic neurons in the brain, releasing the GABAergic tonic inhibition they exert on the IPCs, leading to ILP secretion [85, 86].

The other Akt/PKB well-studied target is FoxO. FoxO is a transcription factor (a family in mammals) that favors catabolism, counteracts anabolism, and is phosphorylated by Akt/PKB to repress its activity [22, 87]. Activation of insulin signaling leads to acute translocation of FoxO proteins out of the nucleus and attenuation of their transcriptional program [88, 89]. In vertebrates, the Forkhead box O (FoxO) family consists of FoxO1, FoxO3, FoxO4, and FoxO6 proteins; a distinct gene encodes each one.* Drosophila* has only one such gene [90, 91]. FoxO proteins bind to the insulin response element (IRE) to stimulate target gene expression on diverse pathways including cell metabolism, proliferation, differentiation, oxidative stress, cell survival, senescence, autophagy, and aging, counteracting insulin action [92].

FoxO repression via insulin signaling activity results in an attenuation of FoxO-dependent expression of genes like those coding for glucose 6-phosphatase or phosphoenolpyruvate carboxykinase [93, 94]. Among other genes, FoxO-regulated antioxidants include the Mn-dependent superoxide dismutase [95]. Signaling pathways that regulate stress and redox status also regulate FoxO proteins, thus, impinge on insulin signaling and the diabetic state: p38, AMP-activated protein kinase (AMPK), among others. The NAD-dependent protein deacetylase sirtuin-1 (Sirt1) directly modifies FoxO transcription factors and promotes their nuclear translocation and activation of target genes [96]. In addition, the acetylation state of histones and the FoxO coactivator PGC-1*α* (peroxisome proliferator-activated receptor *γ*-coactivator-1*α*) may modify the effect of a stimulus on FoxO-induced gene transcription [97]. In conclusion, the FoxO genes transcription is regulated by a variety of physiological cues and pathological stress stimuli frequently associated with increased oxidative stress.

### 1.2. Oxidative Stress and Insulin Signaling

Oxidative stress is considered a key factor in the development and progression of diabetes and its complications [98]. In vertebrates, Sestrins 1–3 (Sesns) form a family of conserved stress-responsive proteins [99]. The Sesns regulate the insulin pathway by regulating the AMP kinase and TOR [100, 101]. Sesn1 was identified as the product of a gene (PA26) activated by the transcription factor p53 in cells exposed to genotoxic stress. Later, it was isolated also as a FoxO responsive gene in growth factor stimulated cells [102]. Sesn 2 promotes the degradation of Kelch-like protein 1 (Keap1) leading to upregulation of Nrf2 signaling and the induction of genes for antioxidant enzymes. The adaptor protein p62 is required for the Sesn 2-dependent activation of Nrf2 [103]. Sesns block TOR-C1 activation and thereby reduce reactive oxygen species accumulation [104]. In* Drosophila*, a single Sestrin homolog has been isolated and characterized. It is activated by accumulation of reactive oxygen species and regulates insulin signaling. Mutant flies suffer from metabolic disarray, muscle wasting, and mitochondrial dysfunction [105]. The* Drosophila *Sestrin acts through two GATOR protein complexes. These GATOR complexes regulate the activity of the RagB GTPase, necessary for TOR-C1 activity. The* Drosophila *Sestrin binds to GATOR2. Bound to Sestrin, GATOR2 frees GATOR1. Free GATOR1 inhibits RagB function by activating its GTPase activity, thus, inhibiting TOR-C1 activation [106, 107]. In vertebrates, the GATOR complexes act in the same fashion; GATOR complexes are evolutionarily conserved in metazoans [108].

Reduction of energy levels in the cells causes the activation of the AMP-activated protein kinase, AMP kinase. This, in its turn, results in TSC2 phosphorylation and subsequent TOR-C1 inhibition. A hypoxic state also reduces TOR activity via the hypoxia-inducible factor-1 (Hif-1) that affects the hypoxia-induced response genes* Redd1 *[109] and* Scylla* [110].* Scylla *forms a complex with* charybdis*, negatively regulating TOR-C1 acting downstream of Akt/PKB and upstream of TSC [110].

## 2. Experimental Animal Models: Vertebrates

Type 1 diabetes is characterized by progressive *β*-cell destruction. Insulin resistance in target tissues characterizes type 2 diabetes. The majority of obese individuals do not become diabetic, although over weight or obesity are clear risk factors for diabetes. In the United States, 87.5% of adults over 18 years old were overweight (including obese and morbidly obese individuals), and an estimated 12.2% of the population is diabetic in 2017 [111], suggesting that *β*-pancreatic cells failure is required to cause hyperglycemia [112]. Due to its overall evolutionary conservation, animal models are used to identify mechanisms, principles, and potential drug targets, besides elucidating general underpinnings of biological metabolic significance. Many animal models of diabetes are currently available for elucidating the pathophysiology of diabetes and testing novel therapies for complications. However, since diabetes etiology is multifactorial, no single animal model may exactly replicate the human situation. Several of these animal models can be used to study chronic diabetes phenotypes.

In principle, all of the models reviewed below could be used for chronic aspects of diabetes and the accruement and evolution of the diabetic state. In spite of this opportunity, in most cases experiments are begun when the diabetic model organisms have advanced to a frank diabetic state (for example, when the resting glucose level is above 250–300 mg/dl several days/weeks after streptozotocin (STZ) injection in rats; see below). It is desirable to study the initial states, starting when the STZ injection is given, and studying the acquirement of the diabetic state, as well as its ulterior evolution. The models reviewed here could well serve or have served this purpose.

### 2.1. Chemical Induction of Diabetes

Alloxan and STZ treatments are the most used diabetes models for diabetic complications in vertebrates. Both chemicals are toxic glucose analogues transported into the cells via the Glut 2 transporter [113]. Both treatments lead to necrosis, importantly of insulin-producing cells, but by different mechanisms. Alloxan generates toxic free radicals, leading to cell death via necrosis. STZ is cleaved, generating free methylnitrosourea that induces DNA fragmentation and necrotic cell decay [114]. Although STZ may also have toxic effects on other organs, its effectiveness and side effects depend mainly on tissue-specific Glut 2 expression, animal age, and nutritional status [114]. STZ administration to 0–2-day-old rats induces an inadequate beta cell mass used as a type 2 diabetes model [115].

A variety of mammals, rodents, rabbits, dogs, pigs, and nonhuman primates, have been used as models of STZ- and alloxan-induced diabetes. The small size of rodents and rabbits results advantageous for maintenance costs, especially in longitudinal studies, but somewhat limits sample material available per animal. In recent years, the pig has gained importance because of its size and close similarity to human physiology. Minipigs have clear advantages over domestic pigs, and genetic modifications leading to diabetic phenotypes have been developed [116].

### 2.2. Genetic Vertebrate Models of Diabetes

Yet to date, rodents represent the predominant vertebrate species used in biomedical research because of traditional use and accumulated knowledge, known animal husbandry, evolutionarily conserved metabolic pathways, the capacity to conduct experiments in organs and study physiology, and, more recently, genetic manipulation possibilities. Among them, several genetic mutant strains are extensively used, depending on the diabetic aspect under study.

The Akita mice have an Ins2+/C96Y mutation, a single nucleotide substitution in the insulin 2 gene (Ins2). This mutation causes reduced insulin secretion, resulting in the development of type 1 diabetes [117]. The db/db mouse is the most popular model of type 2 diabetes. They have a deletion mutation in the leptin receptor resulting in defective receptor function for the adipocyte-derived hormone leptin. This mutation leads to the developing of obesity, insulin resistance, and diabetes [118]. Other mutants with altered metabolism such as the agouti (Ay) mouse, a polygenic model of obesity-induced diabetes, and the ApoE −/− (apoliporotein E deficient) mouse, an atherosclerosis model, are available [119–122].

The Zucker fatty (ZF) rat ports a homozygous missense mutation (fatty, fa) in the leptin receptor gene and develops obesity without diabetes, although rats develop progressive insulin resistance and glucose intolerance [123]. The Wistar fatty (WF) rat is a congenic strain of the Wistar Kyoto rat that also has a fa/fa homozygous missense mutation in the leptin receptor gene. This strain develops obesity [124, 125]. The Otsuka Long-Evans Tokushima Fatty (OLETF) rat is a recognized model of type 2 diabetes. Rats show impaired glucose tolerance, observed from 8 weeks of age, hyperglycemia, and peripheral insulin resistance [126, 127]. The Goto-Kakizaki (GK) rat is a model of nonobese type 2 diabetes. This is a Wistar substrain that develops mild hyperglycemia, insulin resistance, and hiperinsulinemia [128–131]. The ZDF-Lepr*fa*/Crl rat was originated in a colony of Zucker rats, expressing type 2 diabetes, among other models [132–134].

In addition, there are also a variety of different polygenic models of obesity that include the KK-AY mice [135], New Zealand obese (NZO) mice [136], the TALLYHO/Jng mice [137], and the OLETF rats [127], besides diet-induced models of obesity [30]. These models also lead to diabetic states.

## 3. Invertebrate Insulin Signaling

Besides vertebrate diabetes models, two main invertebrate models have been used in experiments. These two invertebrate models are the nematode* Caenorhabditis elegans* and the fruit fly* Drosophila melanogaster*.

### 3.1. *Caenorhabditis elegans*

In the nematode* Caenorhabditis elegans* some of the insulin pathway main components were first characterized, like the nematode homolog,* age-1. *In* C. elegans*, faulty insulin signaling leads to life extension, metabolism changes, disrupted growth, and stress resilience, reminiscent of some diabetic phenotypes [138, 139].

In this nematode the insulin pathway genes were discovered by virtue of their control of dauer larva formation and longevity, evidencing a relationship between aging/nutrition/lifespan [140]. Dauer larvae are formed between larval stages two and three and represent an alternative third larval stage that can survive harsh environmental conditions for up to four months. The pivotal genes in the* C. elegans *insulin pathway are evolutionarily conserved. The insulin receptor homolog is* daf-2 *(from* abnormal dauer formation*), the IRS homolog is* ist-1* [141], the PI3K catalytic subunit is* age-1* (from* aging alteration*), and the regulatory subunit is* aap-1* [141], PTEN is* daf-18*, Akt is Akt-1 and Akt-2, and FoxO is* daf-16* [142]. Similar to the case in* Drosophila*, the insulin pathway is unique and required for many functions including nutritional assessment and metabolism, growth, lifecycle, longevity, and behavior. The study of dauer larvae formation in* C. elegans* has already yielded insights into metabolic/nutritional control and longevity with relevance to humans [143]. There have also been studies regarding behavior modifications, degenerative diseases, and the roles played by FoxO transcription factors in the worm and humans [144]. Learning, memory, and organismal growth are also other chronic conditions where research in* C. elegans* insulin pathway has pinpointed general functions [142].

### 3.2. *Drosophila* and Diabetes

*D. melanogaster* insulin signaling has been evolutionarily conserved, and both types 1 and 2 diabetes can be modeled. Reducing or nearly abrogating expression of the insulin-like peptides (ILP) in the fly can achieve type 1 diabetes [23]. On the other hand, several manipulations can lead to diabetes type 2: mutations in the insulin pathway components downstream from the ILPs [27, 45], dietary manipulations leading to obesity, metabolic imbalance, and hyperglycemia [145–148], or studies in other* Drosophila* species with different lifestyles/diets [149, 150]. As these different experimental protocols, applied in genetically homogeneous fly populations converge, essentially, in a reproducibly diabetic state and faulty insulin signaling, they can all be used for longitudinal studies characterizing the accruement and evolution of compromised diabetic signaling, diabetic phenotypes, and their consequences.

*Drosophila *is uniquely poised to study the insulin pathway and diabetes chronic aspects: it has a very well-developed genetic toolkit simply not available, or not as easily amenable, and with higher genetic background homogeneity and rigor as other models, a very highly polished sequenced genome, a “simplified” insulin pathway, with components exhibiting far less redundancy than, for example, vertebrate models, and the availability of different species with similar sequenced genomes that represent “natural” experiments with different lifestyles and diets, among other advantages. It is particularly of note the capacity to generate different types of genetic mosaics in the whole organism, allowing study and analysis of the cell, tissue, and organismal consequences, and cell independence of mutations, and the localization of functional “foci.” Another related advantage is the possibility of generating space and time limited genetic mosaics that can be used to distinguish between developmental defects versus metabolic defects, for example.

#### 3.2.1. Different* Drosophila* Species Lead to Different Lifestyles

Most of the well-known* Drosophila* species are saprophytic [151]. Inside this big genus (over 2,000 species described so far), there are both generalist and specialist ones, with omnivorous or restricted diets. The environmental conditions that each population faces, together with the availability of nutrients, altitude, latitude, temperature, and so on, can impinge on differences and adaptations that have effects, whether direct or indirect, on insulin signaling. It can affect the levels of activity and, in general, the lifestyle of populations. There are studies examining the effect of varying diets in different* Drosophila* species, whether or not they support life of the organisms in a long-term basis [149, 150, 152]. Some of these changes may or may not have to do with adaptations involving the insulin pathway [153, 154]. In any case, the fact that the genomes plus many other ecological and genomic variables are already known [155–157] implies a great advantage for insulin pathway studies of these ecologically diverse species [158]. This avenue of research represents a window of opportunity as there are more and more* Drosophila* species characterized that can be cultivated in the laboratory, with their genomes sequenced, available for study [159].

Other examples of studies with different* Drosophila* species include* D. simulans*, where metabolic rate, longevity, and resistance to stress have been studied [160, 161].* D. sechellia*, found only in the Seychelles archipelago and requiring the fruit* Morinda citrifolia* as specialized and niche nutrition, toxic for other species, has been thoroughly researched [162, 163]. A recent adaptation of a population of* D. yakuba* to the same nutritional resource as* D. sechellia* in an island population (as opposed to conspecific populations in the continent), namely, Morinda fruit, is striking. This represents a particularly interesting case of a recent adaptation to a major diet shift [164].* D. mojavensis *requires cacti as a feeding resource and has even specialized to different host cacti in different populations [165]. Together, they may allow dissection of the mechanisms behind the differences and preferences for specific nutrients, oviposition sites, and their tolerance and metabolism. For example,* D. mojavensis* shows a better resistance to the presence of alcohol, a product of the fermentation of cacti [166]. It will be interesting to study in these examples the changes, if any, in the insulin pathway due to their specialized and restricted nutritional resources, and how might a diabetic state alter their metabolism.

#### 3.2.2. *Drosophila melanogaster* and Insulin Signaling

In* D. melanogaster*, the growth of the organism is regulated by insulin signaling and the interaction of this signal with the levels of juvenile hormone and ecdysone. Additionally, there is the role played by the kinase TOR of the insulin pathway, which, as stated above, couples growth with the amount of available nutrients [167], at least in part via the amino acid transporter Slimfast [84]. Since the fruit fly is a poikilothermic organism, it is affected by ambient temperature in a direct way, presenting a larger size at lower temperatures, and an increase in size with latitude and altitude [168]. The number of ovarioles in females is also susceptible to these factors, being lower in tropical populations and it has been shown that insulin signaling activity underlies these differences [169, 170].

Besides growth hormones, diet, and temperature, gut microbiota can modulate insulin pathway activity. Between populations and lines there are differences in the microbiome, and this influences insulin signaling [171, 172]. Strains infected with the* Wolbachia* endosymbiont exhibit increased insulin signaling, whereas lack of* Wolbachia* worsens insulin mutants phenotypes, particularly the decline in fecundity and adult weight [173]. Loss of ILPs in the brain, on the other hand, extends lifespan if* Wolbachia* is present [24].* Lactobacillus* partially rescues growth in poorly fed larvae and* Acetobacter pomorum* also modulates insulin signaling [174, 175]. All of these factors have to be taken into consideration, ideally, when longitudinal studies are performed, since many of these factors may vary with age, independent of the status of the fly.

Longevity has also been tied at times with insulin signaling [176–178]. Experimental model organism lines that were selected because of their increased lifespan, often present changes in the insulin pathway. Hoffman et al. [179] performed WGS and GWAS studies on long-lived* Drosophila* strains and found that the metabolites that decline with age are associated with glycolysis and the metabolism of glycophospholipids. Changes were observed associated with age and sex in biogenic amines, and carnitines, required for the transfer of fatty acids in the mitochondria where they pass through beta-oxidation generating acetyl-coA required for the Krebs cycle.

#### 3.2.3. Inducing the Diabetic State through Diet

Providing* Drosophila* diets with increased or decreased nutrients provokes the deregulation of its metabolism and of insulin signaling. High-sugar as well as high-protein diets increase insulin-like peptide expression (ILPs) [148, 180]; this initial increase in ILP expression is consistent with what is observed in vertebrates in the accruement of insulin resistance, where the organism initially tries to increase its insulin production to compensate for excess nutrient input. However, in vertebrates, the eventual deterioration of beta cells leads to ultimate failure of this initial compensation [181]. Similarly, in overfed flies, the fat body secondarily reduces its insulin response to increased circulating ILPs, and this diminution decreases significantly as flies age, rendering flies completely resistant at advanced ages [147]. These results support the observation that a diet rich in fat initially increases levels of different ILPs, rescuing at a first stage the overfed phenotype by means of hyperinsulinemia. This increase in insulin signaling, plus hyperglycemia, though, leads to an increase in free fatty acids by inappropriate lipolysis and the generation of insulin resistance, particularly in the fat body. High fat diets also contribute to heart dysfunction [145, 182].

Sugar, lipid, and protein variation in diets have led to effects in fertility, longevity, sugar and fat accumulation, weight changes, induction of insulin resistance, and aging [147, 183]. In general, the results are consistent with protein and carbohydrate balance determining lifespan. In some cases diets high in carbohydrates and low in proteins allow greater longevity, often accompanied by lower fecundity. In other studies, extra protein intake results in lean and longer lived flies, while carbohydrate intake leads to obese flies; finally, balanced, intermediate carbohydrate:protein ratios diets have also been found to lead to longer-lived flies [183–187].

Dietary restriction has been variously applied to flies, often leading to longer life-spans and insulin signaling involvement [188–190]. Dietary restriction effects are also seen in immunity via insulin signaling regulation [191]. The insulin signaling is clearly part of the equation in all these studies, although it may not be the sole determinant [192, 193]. One such type of dietary restriction is caloric restriction. Caloric restriction is defined as a reduction in caloric intake without malnutrition and has shown in several models a positive effect on calorie consumption, including yeast and* C. elegans* besides* D. melanogaster* [194–196]. Another variation of diet manipulation with nutritional and lifespan consequences is methionine availability in the diet [197]. Also, parental obesity leads to transgenerational effects [198].

Clearly, diet manipulation can be used to generate and evolve diabetic states akin to diabetes type 2 in flies, and by its very nature, it is easily amenable for longitudinal studies. Yet an important problem besieging all these studies, and one that may explain conflicting results, is that all these diet regimes are semidefined chemically, at best, so that studies that modify diets are very difficult to compare. They typically define “protein” as amount of yeast in the medium, or “carbohydrate” as unrefined sugar or molasses, for example, which are clearly broad generalizations, as yeast cells have carbohydrates, lipids, and other nutrients besides proteins, and unrefined sugar or molasses are not only composed of carbohydrates. In the future, diets should strive to be defined chemically, so that they can be comparable and used reproducibly by different laboratories. In addition, total consumption should be measured, since unconstrained flies have free access to their food source and are able to regulate their caloric intake, at least in the case of* D. melanogaster* [183]. Also, these studies are subject to environmental variations, the use of different sexes, different genetic backgrounds, different strains, different age of flies, and so on, all of which affect the outcome of the studies. And while these studies show that changes in diet have clear effects on the body and insulin signaling, lack of definition constitutes a limiting factor in these experimental approaches.

#### 3.2.4. Diabetes in Flies by Virtue of Mutations in the Insulin Pathway

Flies homozygous mutant for genes in the insulin pathway are born diabetic. There are advantages to this approach: the nature of the defect is known, and the genetic background and environmental conditions can be controlled in a rigorous manner. The different stages of the lifecycle can also be exploited, with stages where feeding occurs (larvae, adults), and stages without food input (pupae). Faulty insulin signaling by virtue of mutations can both be used to model diabetes type 1 (ILPs loss-of-function mutations [23], and even explore genes regulating insulin secretion [199]), and also diabetes type 2, with loss-of-function mutations in the rest of the pathway, as the net effect would be insulin resistance [27, 31, 71]. Furthermore, both whole organisms can be mutant (in hypomorphic conditions, as null alleles are mostly lethal), or only in selected tissues and organs ([18, 45, 48, 77, 91, 200, 201], among other references cited throughout this review).

*Drosophila *has seven insulin-like peptides (ILPs). They are partially redundant, so knock-outs for a particular ILP usually have moderate effects, and it is necessary to have more than one ILP gene loss-of-function mutation to generate lethality [23, 24]. In contrast, mutations in* InR, Dp110, *and other components of the pathway are homozygous lethal, so heterozygous (as there are some dominant effects [45, 202]) or heteroallelic flies are often used. The latter have the benefit of allocating more robustly the defects observed to the studied mutation (for example, see [31]).* chico* (the insulin receptor substrate fly homolog) is a particular case. The homozygous mutant* chico*^1^ allele was originally described as viable, helping establish the typical mutant phenotypes of partial loss-of-function of insulin pathway mutants [45]. Later it was found out that, when devoid of the endosymbiont* Wolbachia*,* chico* loss-of-function conditions are homozygous lethal, underscoring the close association between gut microbiota and insulin signaling [173].

The most common phenotypes caused by mutants in the insulin pathway are a decrease in fertility, decreased size of organisms, changes in longevity (decrease in normal conditions, often an increase in longevity when there is caloric restriction), defects in fat body morphology, in heart, retina, and brain physiology, increased levels of triacylglycerides, and higher amounts of circulating sugars in the hemolymph [31, 45, 71, 145, 147, 203–205].

Insulin/TOR pathway function is critical in the regulation of growth, autophagy, cell and organism survival, and anabolism (regulating lipid and carbohydrate homeostasis) [22]. Lack of nutrients or ATP impedes its function, and overfeeding can lead to loss of balance: insulin participates in the accumulation of lipids and carbohydrates, so that an excessive intake of nutrients can lead to hyperactivation of the pathway, and lipid and glycogen accumulation. TOR kinase is regulated by insulin signaling and by amino acids, acting as a central point in metabolism regulation. It has also been implicated in aging [206–208].

Besides nutritional input, there are other conditions that regulate insulin signaling: as an example, one such state is the systemic response to stress (which, of course, is activated by lack of nutrition, among other stimuli). One such stress trigger is infection, and innate immunity activation. Activation of Toll in the fat body leads to the induction of immunity, redistribution of resources, and activation of JNK and NFK-*β* by inflammation. Here, attenuation of insulin signaling leads to FoxO activity, which regulates genes participating in stress response and metabolic control: blockade of gluconeogenesis, glycogenolysis, and the use of storage lipids for catabolism [209]. It may also increase longevity if FoxO is upregulated in adipose or intestinal tissue [210]. In addition, chronic intestinal activation of FoxO may lead to deregulation of lipid homeostasis [211].

#### 3.2.5. Towards the Characterization of Chronic Diabetes in Flies

What can be studied in these fly diabetes models in a longitudinal study? Nearly every aspect of diabetes mellitus mentioned so far: from initial phenotypes, to its evolution and consequences at old age, up to death. We have discussed above metabolic imbalances and longevity as two of the most studied effects [212, 213]. Other mutant phenotypes include decreased fertility and altered physiology of various organs and systems (nervous system, heart, fat tissue, muscles, etc.), for example, showing involvement of nervous system function: electrical activity or octopamine neurotransmission [214, 215] or heart dysfunction [216].

Perturbations like chronic stress can be addressed, and the effects of external factors on the diabetic state, such as nutritional variation, light regime, and temperature, can also be addressed, for example, the effect of artificial sweeteners upon insulin pathway signaling [217] or the effect of xenobiotics on the insulin pathway [218]. Fundamentally, though, the inception and “normal” progression of the mutant condition or the diseased state can be closely followed, for example, the relationship between sleep and metabolism alterations via insulin signaling [219–221]. Unfortunately, to this day few studies have consciously addressed these chronic aspects of diabetes, although some do compare flies at different stages, like during oogenesis [222], or pupariation [223], or neurite remodeling [224], or adult stages [225].

Most of the studies to date that touch on longitudinal aspects address longevity, fertility [226], and its phenocritical period [23, 202, 227, 228], like the earlier appearance of locomotor defects [229], size and growth phenotypes [45, 200, 201, 230], and metabolism [71]. In summary, the* Drosophila* model represents a window of opportunity not only to study fundamental aspects of diabetes and the diabetic state, but also its complications, and the effect of various external stimuli and factors in the accruement and development of the disease. Future studies will, undoubtedly, target and address these issues to a much greater extent.

## 4. Conclusions

Notwithstanding the ubiquity and utility of vertebrate models of diabetes (especially rodent models), and when compared with other experimental models of diabetes,* Drosophila* has clear advantages. Despite sometimes ill-defined parameters in diet regimes and environment, the model strengths, namely, a robust, extensive, and highly developed genetic system, with a plethora of isolated and characterized insulin pathway and general metabolism genes, ease of manipulation and use, low cost, high fertility, and numbers, short generation time, high evolutionary conservation, and homogeneous genetic backgrounds, among other positive characteristics, make the fly a premier system for insulin pathway and metabolic studies.

Although many aspects of diabetes mellitus have been studied in the different fly models specially, there is still a dearth of longitudinal studies. In such studies, ideally exception should be taken of the differences that occur in metabolism and lifestyle normally as flies age; that is to say, these studies should always par appropriate control flies with experimental ones throughout the life cycle with the same genetic background, to effectively tease away differences due to the occurrence and evolution of the diabetic state from normal aging. This is especially true of a disease that touches many different aspects of the organisms' wellbeing, and one that by its very nature is very pleiotropic and polygenic.

Notwithstanding this, the fruit fly represents one of the more promising, rigorous, and thoroughly researched models of diabetes in which we carry out such research. Due to great evolutionary conservation, it allows for particularly detailed and controlled studies covering nearly all aspects of this chronic and fatal disease. Compared to other available models, that is, vertebrate studies, be it organismal or even cell tissue culture ones, the fly favorably compares, allowing for more holistic and encompassing approaches.
